# Methods for numerical simulation of soft actively contractile materials

**DOI:** 10.1038/s41598-023-36465-x

**Published:** 2023-06-26

**Authors:** Yali Li, Nakhiah C. Goulbourne

**Affiliations:** University of Michigan, Ann Arbor, USA

**Keywords:** Mechanical engineering, Soft materials

## Abstract

Soft materials that can demonstrate on demand reconfigurability and changing compliance are highly sought after as actuator materials in many fields such as soft robotics and biotechnology. Whilst there are numerous proof of concept materials and devices, rigorous predictive models of deformation have not been well-established or widely adopted. In this paper, we discuss programming complex three-dimensional deformations of a soft intrinsically anisotropic material by controlling the orientation of the contractile units and/or direction of the applied electric field. Programming is achieved by patterning contractile units and/or selectively activating spatial regions. A new constitutive model is derived to describe the soft intrinsic anisotropy of soft materials. The model is developed within a continuum mechanics framework using an invariant-based formulation. Computational implementation allows us to simulate the complex three-dimensional shape response when activated by electric field. Several examples of the achievable Gauss-curved surfaces are demonstrated. Our computational analysis introduces a mechanics-based framework for design when considering soft morphing materials with intrinsic anisotropy, and is meant to inspire the development of new soft active materials.

## Introduction

Electrically-activated elastomeric materials hold great promise to push the frontiers of autonomous life-like soft robots^1,2^. Fast actuation of materials that match the compliance and rheology of biological materials could enable breakthroughs in realistically mimicking the rippling fin motion of a cuttlefish, the dynamic acrobatics and dexterity of a bat wing skin during flight^3–6^, and even simulate the contractions of arteries^7^ and pumping motion of the left ventricle of the heart^8^. Unlike their rigid counterparts, soft robotics could enable future breakthroughs in physical human–robot interactions, performing tasks in unstructured environments not suitable for humans, and as implants interacting with living soft tissue^9–11^. To meet this need, there have been several soft materials proposed to date ranging from very soft hydrogels to shape memory polymers whose shape change can be triggered by heat, magnetic field, electric field, and light^12^.

Of the soft active materials investigated to date, dielectric elastomers (DEs) have garnered significant interest^13,14^ due to their large and reversible fast deformations in response to an applied electric field. Classically, dielectric elastomer actuators are made by sandwiching a thin prestretched polyacrylate film in between two carbon grease electrodes^15–17^. The main drawback limiting wide-spread adoption is the large magnitude of the electric field, the prestrain requirement, and their rather low force output in comparison to stiffer active materials. Various solutions and mechanisms have been proposed to leverage their strengths and minimize drawbacks. Several groups have investigated mechanisms for adding stiffer fibers, for example, increasing the dielectric constant of the polymer, replacing carbon grease with more stable electrodes, and patterning the electrodes^18–28^.

Another soft elastomer demonstrating large deformations are liquid crystal elastomers (LCEs) triggered by light or temperature near their phase transition temperature. Electric actuation is much more attractive for robotic control and unfortunately the electro-mechanical strains of LCEs are reportedly small compared to that of DEs. Recently, Davidson et al. demonstrated the ability to spatially tailor the pattern of LCE molecules and increase the efficiency and strains of a dielectric LCE actuator^29^. A dielectric LCE actuator is one that is configured as a traditional dielectric elastomer actuator using a LCE as the dielectric. Starting with the typical planar capacitor configuration of a DE and noting that they undergo large area expansion and thickness reduction when an electric field is applied between the two soft electrodes sandwiching the dielectric, Davidson et al. replaced the rubbery elastomer with a molecularly aligned LCE. By using a top-down photo-alignment method they created large localized elastic anisotropy into the LCE. This is an example of *material anisotropy* or *intrinsic anisotropy* i.e. the macroscopically observed anisotropy is due to molecular scale ordering and localized elastic anisotropy. Unlike typical LCEs, their work demonstrated direct electric field actuation of LCEs whereby contraction is achieved by exploiting the large local mechanical anisotropy of LCEs and without relying on local molecular rotation. Prospects for implementing dielectric LCEs in soft robotics are promising although several challenges remain^30^. The intrinsic anisotropy of LCEs is in sharp contrast to the prior mentioned practice of incorporating stiff fibers into an elastomer or patterning compliant electrodes locally and spatially when fabricating traditional dielectric elastomers, which are examples of *mechanical anisotropy or extrinsic anisotropy*. Using either approach (intrinsic anisotropy or mechanical anisotropy), inhomogeneous deformations and complex 3D shapes can be obtained when an electric field is applied. For example, it has been demonstrated that DE circular sheets will deform into conical sheets by adding and actuating stiff concentric electrode rings patterned into elastomer layers^28^. Notwithstanding these results, the necessary soft active materials for biologically similar soft robotic actuation do not exist.

A close look at the microstructure of nature’s soft active materials reveals a consistent theme of soft intrinsic anisotropy. Examples include smooth muscles in the artery wall that contract and relax to change the volume and pressure of blood vessels^8^, the discrete musculature found in bat wing membranes that actively vary local compliance of the wing for benefits in aerodynamic performance^3,4^, and the muscular hydrostats in the cuttlefish fin and ring muscles in jellyfish that enable swimming performance^31^. Inspired by biology and various engineering attempts to program rubbery dielectrics into 3D shapes, we present an overarching framework that can be used to simulate, explore, and predict the shape change of an electrically activated soft material with intrinsic anisotropy. We generalize the problem of predicting the 3D shape change of a soft elastomer to now account for embedded contractile units (we conceptualize these as molecularly aligned units or ‘microscopically oriented fibers’ modeled as analogs to biological muscle units). We employ a finite deformation computational framework for modeling soft electro-responsive anisotropic materials. The framework incorporates nonlinear viscoelastic effects using Reese and Govindjee’s multiplicative decomposition of the deformation gradient into elastic and viscous parts^32^. This model was initially introduced in the context of material inhomogeneities^33^ and finite elasto-plasticity and has since been widely adopted by the mechanics community, for example, in modeling biological growth and adaptation^34,35^. The computational simulations point to several new opportunities for the design, synthesis, and fabrication of novel materials and material architectures to generate complex three dimensional deformations.

In this work, we highlight novel mechanisms that could be exploited to develop new soft robotic materials by introducing three new concepts. The first is the concept of dual mode activation via matrix activation and ‘fiber’ activation. We later drop the quotes on ‘fiber’ as it is understood that by fiber activation we are referring to the molecular-scale ordering of contractile units in the material. Activating the matrix and/or contractile units renders a larger range of tailorable configurations and hence performance than previously achievable by either isotropic or passively anisotropic counterparts. The second concept demonstrates a surface twisting mode due to intrinsic anisotropy. The third concept combines sequential and spatial activation for 3D shape patterning, which is demonstrated with a rectangular membrane example. The role of directionality in influencing membrane shape is emphasized.

## Materials and modeling methods

All of the work is computational in nature. We developed a constitutive model to describe electromechanical coupling in soft anisotropic materials undergoing large deformations. Nonlinear viscoelastic effects are accounted for in a thermodynamically consistent way. In the constitutive model, we introduce an invariant-based strain energy function as an additive combination of purely mechanical and electromechanical contributions. The mechanical part captures the elastic and viscous behavior and the electromechanical part captures the coupling response. Here, the constitutive equations are particularized assuming a quadratic dependence on the electric field (the activation), which is in addition to the electrostatic coupling of the matrix (reminiscent of the electrostatic coupling in dielectric elastomers). The coupled constitutive formulation highlights a new electromechanical coupling term to capture the field-based response of the contractile units. This intrinsic (or active) anisotropy alters the electromechanical response by adding an initial offset to the total Cauchy stress. The constitutive response can be tuned depending on the electric field direction or alignment of the contractile units, which creates a vast materials design space based on intrinsic orientations. From this more general formulation, we can easily recover known constitutive formulations for isotropic and passively anisotropic dielectric elastomers^36,37^. We implement the constitutive model into a finite element solver by prescribing a user-defined material with the user subroutine UMAT in ABAQUS. The constitutive model is outlined in the Supplementary Material alongside the necessary equations for computational implementation in a commercial finite element solver. The total Cauchy stress tensor is directly coded and we define the internal variable of the evolution equation through state variables in the UMAT. The evolution equation is solved by the Euler forward difference method in explicit form. The evolution equation can alternately be formulated with the Euler backward difference method in implicit form and solved using Newton’s method. Since the tangent modulus does not affect the accuracy of the solution but the rate of convergence when solving the residual equations, the tangent modulus is derived using only the mechanical part of the strain energy function (or the second Piola–Kirchhoff stress). We can obtain analytical solutions for only the simplest of cases. Computational implementation allows us to solve more challenging boundary value problems and explore three dimensional deformations of complex shapes for which there are no analytical solutions. The computational implementation of the model is compared with analytical results for a set of simple cases, and the match is excellent. This computational tool can be used to explore a wide range of material models, material architectures, and actuator designs. For example, the constitutive model could be further particularized to capture more complex kinematics of the contractile units, consider a statistical distribution of contractile unit orientation, or even explore heterogeneity due to more than one type of contractile unit. Being embedded in a commercial solver, various designs (geometry and material combinations) can easily be explored.

### Materials

Whilst a traditional dielectric elastomer will always expand when actuated, we show in this paper that intrinsic contractile units (at the molecular or mesoscale) will yield muscle-like contractile behavior at the macroscale. Furthermore, we demonstrate how coupling electrostatic actuation (Maxwell stress behavior) to intrinsic in plane actuation creates an interesting interplay to program various complex shapes when the material is actuated. This is the first set of simulations exploring the proposed material concept. For the simulations, we select materials with dielectric and material coefficients similar to elastomers commonly used as dielectric elastomer actuators. Silicone and VHB 4910 have values ranging from 2.7 to 4.7. The electric field values are also contained within range of existing materials i.e. up to 12 MV/m. The contractile units are inspired by biological muscle fibers and modeled analogously i.e. we introduce a term in the strain energy function that is proportional to the square of the fiber stretch (invariant ${I}_{4}$) through a new electromechanical coupling coefficient. We explore the dynamics of the ratio between fiber and matrix material properties but conservatively keep the properties within range of known materials. One way that the contractile units could be realized is by arranging a series of dielectric elastomer discs in a stacked configuration to form embedded fibers. Another way would be to pattern molecular scale ordering into dielectric liquid crystal elastomer actuators. More recently, synthetic biology and advances in biomaterial sciences has given rise to engineered living materials, which consist of living cells and/or living organisms in synthetic polymer matrices like hydrogels. The living cells are active and functional constituents within the host synthetic material^1,38,39^. Programming mammalian muscle cells and organisms such as algae, bacteria, and fungi for the next generation of biomaterials is currently being explored. Whilst still in its infancy, biological contractile cells as engineered living materials might soon be within reach as future stimuli responsive materials^40–46^. Other methods might be motivated by this simulation work.

## Results

In this section, we present a range of computational simulations to illustrate three novel actuation concepts. As mentioned prior, the first is the concept of independent matrix activation and fiber activation that demonstrates dual mode activation with a single design. The second concept demonstrates a surface twisting mode due to fiber anisotropy and contractility. The third concept combines sequential and spatially distributed activation to achieve 3D shape patterning of a rectangular membrane pinned along two edges.

### Basic actuation architecture: membrane and fiber activation

**Figure 1:**
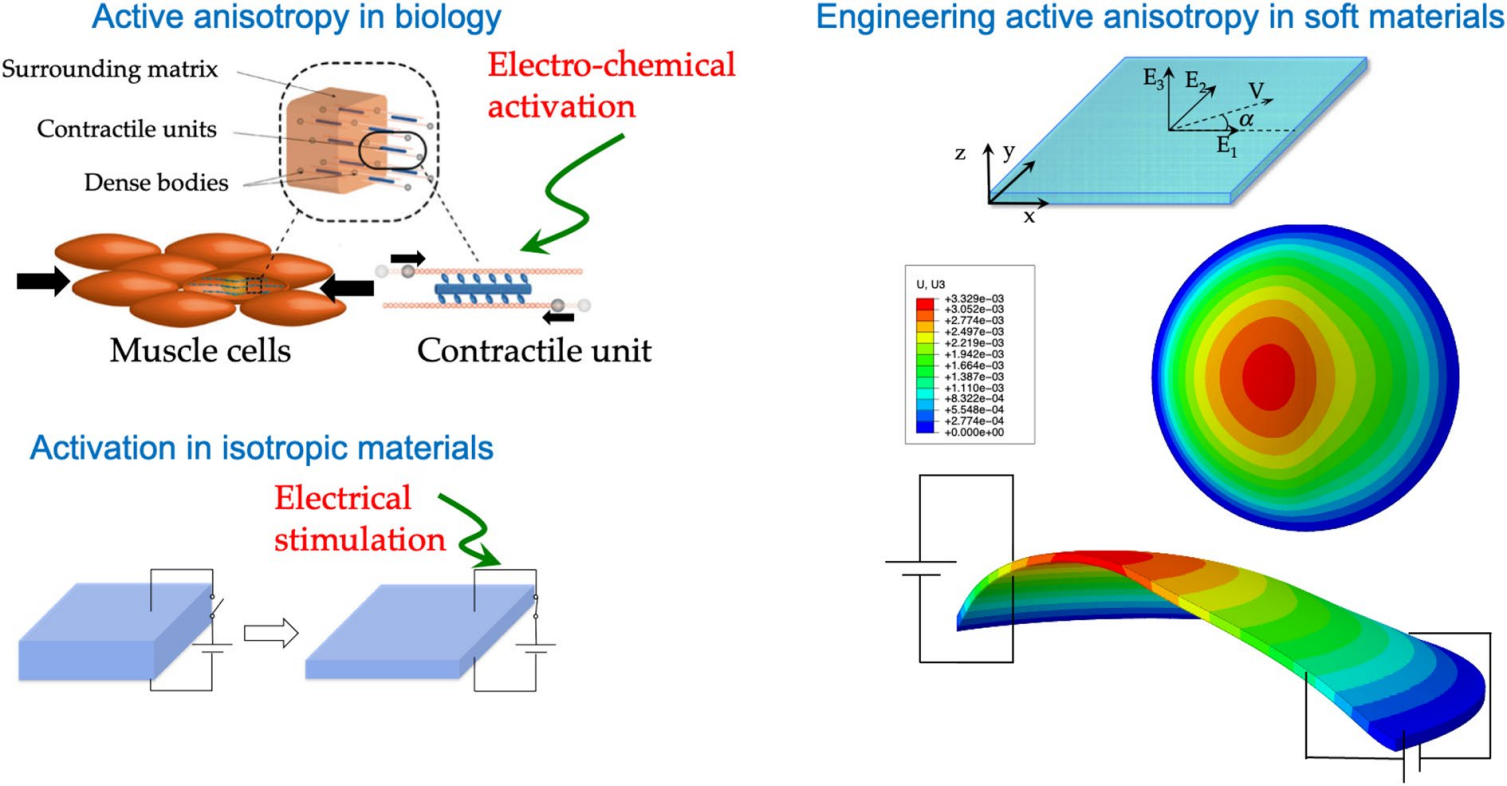
Engineering active anisotropy. (**a**) Schematic of active anisotropy in biology in the contractile muscle unit. (**b**) Schematic of a typical dielectric elastomer actuator in off and on states. (**c**) Schematic of active anisotropy in engineered soft materials.

**Figure 2:**
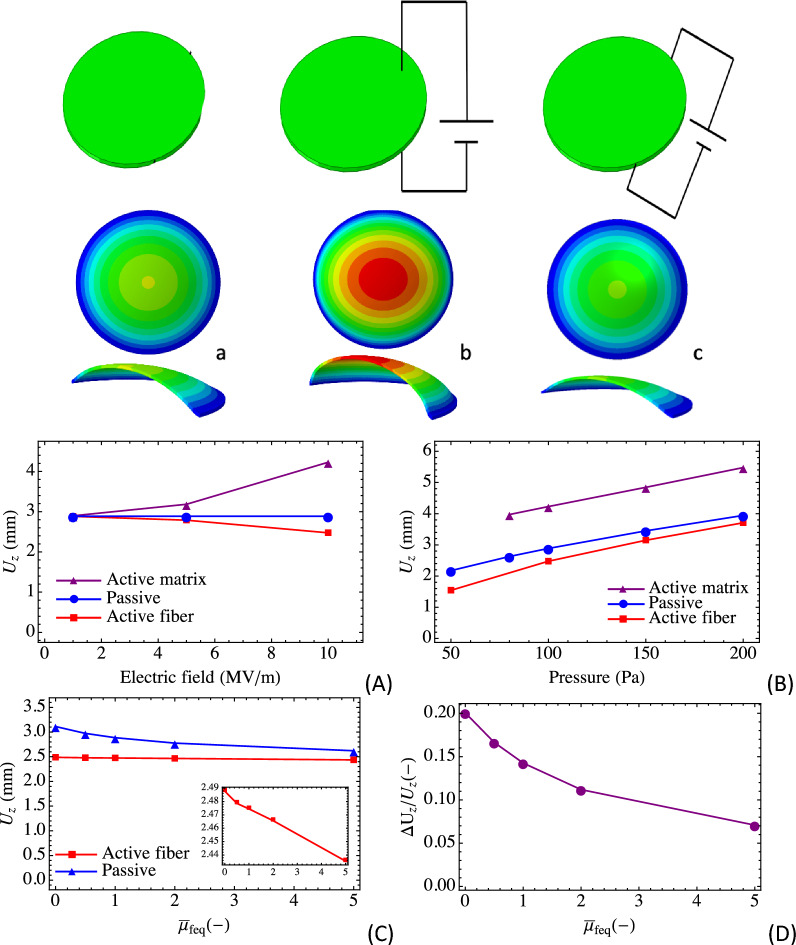
Basic actuation architecture: (**a**) Mechanical pressure loading on a soft material (unactivated), (**b**) matrix activation, (**c**) fiber activation. Plots show the relationship between out of plane peak deflection and (**A**) electric field, and (**B**) external pressure. Plot (**C**) shows the relationship between the out of plane peak deflection and the fiber:matrix modulus ratio. Plot (**D**) shows the relationship between the out of plane peak deflection normalized by the passive peak deflection and the fiber:matrix modulus ratio.

For the computational simulations, we start by considering an intrinsically anisotropic circular elastomer membrane that is pinned around its circumference and subjected to a bias inflation pressure (100 Pa on the bottom surface). A traditional isotropic dielectric elastomer that is constrained around its circumference and subjected to a bias pressure will inflate further when activated (by electric field). If it contains stiff passive fibers then the inflation will be reduced and depending on the fiber distribution alternate surface inflation shapes can be achieved. For example, a circular sheet with passive stiff concentric fibers will inflate as a cone^28^. The membrane diameter is 20 mm and the membrane thickness is 0.5 mm. The material parameters used in the numerical simulations are listed in Table 1, where $\mu$ is the shear modulus of the matrix, ${\mu }_{feq}$ and ${\mu }_{fneq}$ are the moduli for the equilibrium and nonequilibrium parts of the fiber modulus part, $\eta$ is a viscous parameter, ${\varepsilon }_{rf}$ and ${\varepsilon }_{rm}$ are the fiber and matrix dielectric constants, ${\varepsilon }_{0}$ is vacuum permittivity. An overbar denotes a normalized quantity. The fiber moduli are normalized by dividing by the shear modulus, and the viscous parameter is normalized by dividing by four times the nonequilibrium fiber modulus. Within a rectangular coordinate system, the contractile dielectric elastomer fibers are oriented in the y-direction (Fig. 1). The circular membrane is meshed into 40,460 hybrid 8-node brick elements with three elements through the thickness (sufficient per mesh convergence studies). We calculate the response for two types of electric field activation: (i) fiber activation of 12 MV/m and (ii) matrix activation of 10 MV/m, and compare these results with results for the passive (purely mechanical) case. Figure 2 illustrates the two basic modes of actuation: comparing with the passive case, we observe that matrix activation increases the out of plane deflection whilst fiber activation reduces the out of plane deflection—this is dual mode activation. Recall that typical dielectric elastomers will only inflate when activated, whereas recent reports show that molecularly aligned dielectric LCEs will contract like muscle. Figure 2A,B show the out of plane peak deflections for various electric fields and pressure loadings for both types of activations. We observe that there is a nonlinear relationship between the electric field/pressure and the out of plane peak deflection, which is to be expected given the nonlinear material coupling. From the results we can also deduce the effect of the relative material stiffness on the deflection response. Figure 2C,D show that: (i) for an isotropic material the deflection offset relative to the passive case can reach 20% (fiber modulus is zero), and (ii) increasing the stiffness of the fibers decreases the deflection offset (10% for a fiber modulus 5 times the matrix modulus). The influence is modest for the parameter values chosen in the simulation. Looking at the inset of Fig. 2C, we see that the out of plane deflections for the active case decreases with increasing fiber modulus, however only slightly.

**Table 1:** Initial numerical values of model material parameters.

Parameters	$\mu$ (kPa)	${\overline{\mu }}_{feq}$ (–)	$\overline{\mu }_{fneq}$ (–)	$\overline{\eta }$(s)	$\varepsilon_{0}$ (F/m)	$\varepsilon_{mr}$ (–)	$\varepsilon_{fr} /\varepsilon_{mr}$ (–)
Values	10	1	1	0.25	8.85E−12	4.7	2.7/4.7

### Complex and asymmetric shape morphing

#### Simple spatial activation

**Figure 3:**
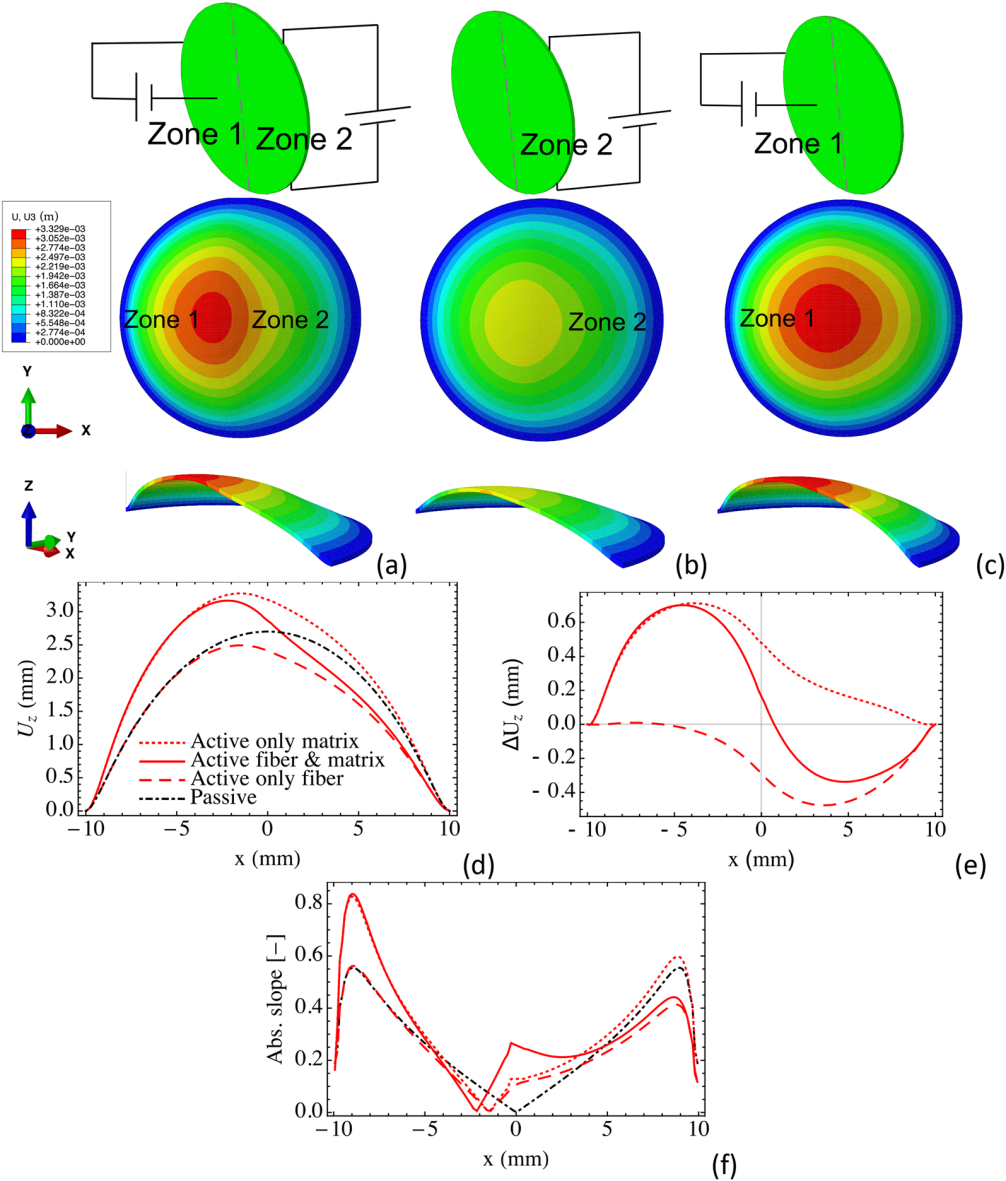
Designing three activation protocols: (**a**) matrix activation in zone 1 and fiber activation in zone 2, (**b**) fiber activation in zone 2 only, and (**c**) matrix activation in zone 1 only. Deformation fields for (**a**) activation in matrix and fibers, (**b**) activation only in fibers, and (**c**) activation only in the matrix. Centerline deflection profiles are calculated in (**d**–**f**): (**d**) Transverse deflection of the horizontal centerlines of the three active cases and the passive case, (**b**) comparison of the deflection offset (relative to the passive case) for the three active cases, and (**c**) the slopes of the horizontal centerlines.

To probe the parameter space of 3D deformations, we demonstrate shape changes, both magnitude and slope, through spatial activation and fiber orientation. We start with the same circular membrane as before with molecularly aligned contractile units oriented in the y-direction. The circumferential edge of the circular membrane is pinned and an offset pressure of 100 Pa is applied to the bottom surface. The membrane is then divided into 2 zones, which are activated according to different protocols. Figure 3 shows three activation protocols: (i) zone 1 activation in the matrix and zone 2 fiber activation, (ii) only zone 2 fiber activation, and (iii) only zone 1 matrix activation. The applied electric field magnitudes for the fibers and matrix are 10 MV/m and 8 MV/m, respectively. We show deformation fields for the three activation cases and plot transverse deflections for the horizontal centerlines relative to the passive case (Fig. 3). Generally, we see that all three activation approaches break the symmetry. Matrix activation creates the largest out of plane deflection whilst fiber activation has the smallest out of plane deflection. Figure 3d shows that each activation protocol shifts the peak deflection value to around 40–45% of the horizontal centerlines, which also can be seen in Fig. 3f when the slope is zero. Figure 3e gives another view of how the deflection magnitude changes for each of the three activation cases. Matrix activation increases the deflection magnitude, for example, at x = 0 the deflection increment is 0.47 mm. Fiber activation decreases the deflection magnitude, for example, at x = 0 the deflection reduction is 0.29 mm. Active both in fibers and matrix yield a peak deflection increment of 0.16 mm at x = 0. Interestingly, this simple zone activation model highlights how we can use active anisotropy to change curvature. Around the center region, the magnitudes of the slopes increases with activation to 0.27, 0.13, and 0.1 for each of the protocols, i.e. the surfaces tilt to 15°, 7.3°, and 5.8°. So with a single initial design, we can program the surface to take on various conformations by controlling the deflection magnitude, surface slope, and peak position (breaking symmetry).

### Effect of molecular scale ordering: orientation

**Figure 4:**
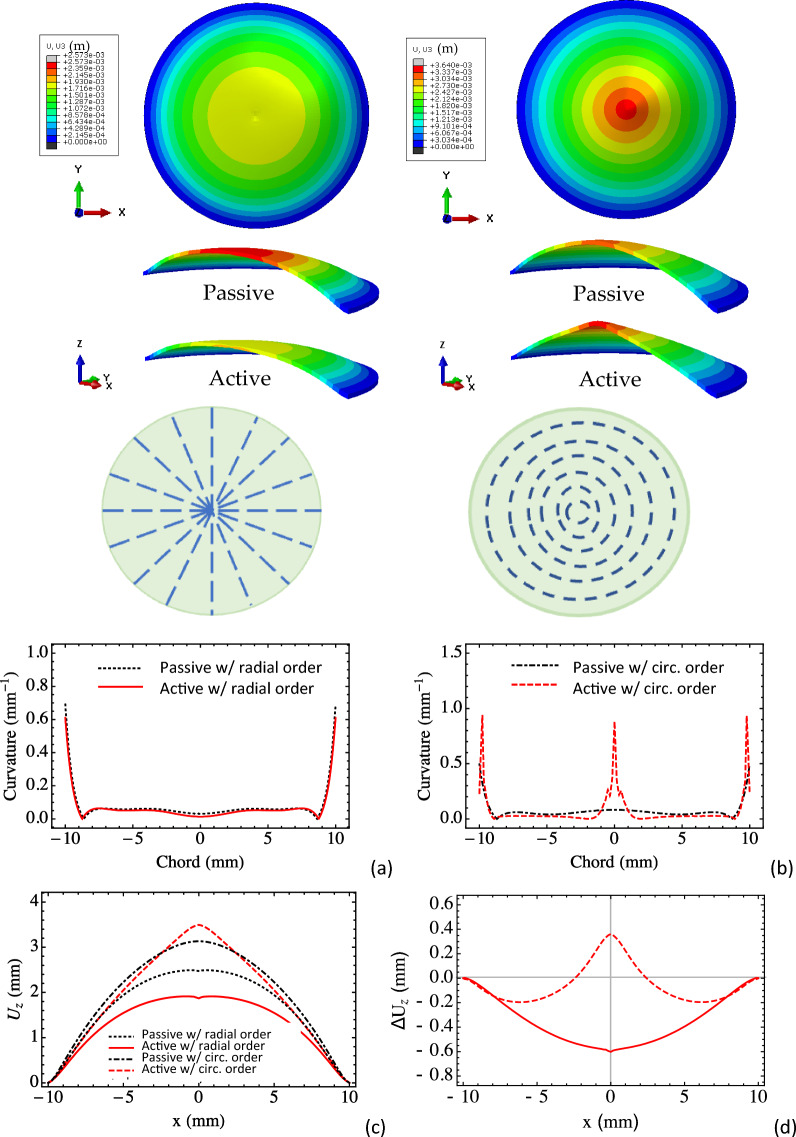
Deformation fields and centerline curvatures for (**a**) radial and (**b**) circumferential order distributions. (**c**) Transverse deflection of the centerline and (**d**) transverse deflection offset by the passive case.

We extend the simulations and consider radial and circumferential order distributions. The magnitudes of the electric field in the contractile units are 12 MV/m for both cases. The deformation fields of the two designs are shown in Fig. 4, and we plot the transverse deflections for the horizontal centerlines to compare and assess them qualitatively. As expected, the deformation fields are axisymmetric and positions of the peak deflections are in the center. However, due to differences in the intrinsic orientations (anisotropy), the activation of the circular membrane with radial order decreases the peak transverse deflection whilst the activation of the circular membrane with circumferential order increases the peak transverse deflection around the center region while the peripheral region has reduced transverse deflection, which leads to the slope and curvature changes of the membrane surface. For circumferential architecture, the membrane deforms into a cone shape and for the radial architecture it deforms as a spherical cap upon activation. Figure 4a,b shows the curvatures for the deformed centerlines. As can be seen, the activation of the membrane with circumferential order alters the curvature of the surface in the center region significantly. While for the membrane with radial order, the curvatures only change slightly between the passive and active cases. (The large curvature around the edge (i.e. at x = − 10 and 10 mm) is due to the pinned boundary condition.)

**Figure 5:**
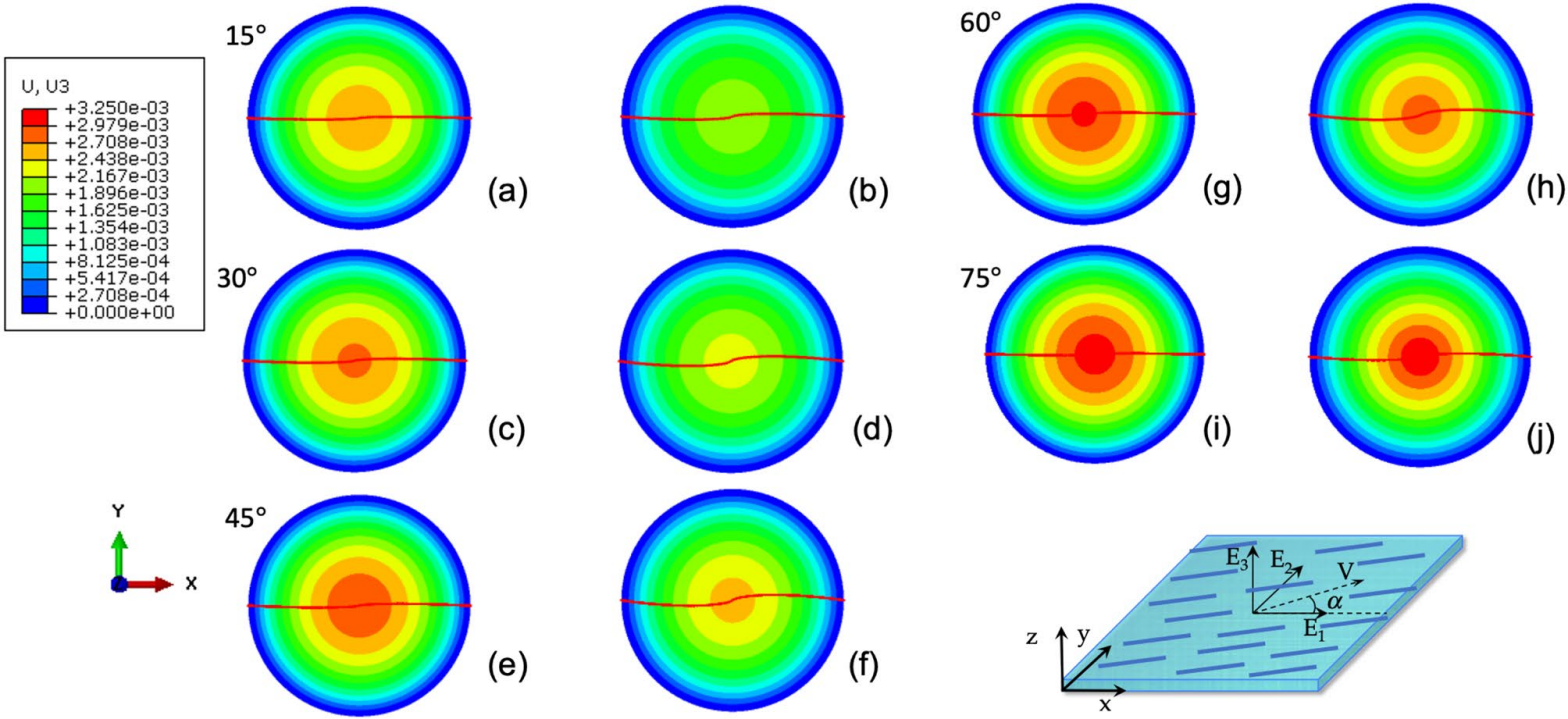
Deformed passive and active configurations for the circular membrane with (**a**,**b**) 15° orientation, (**c**,**d**) 30° orientation, (**e**,**f**) 45° orientation, (**g**,**h**) 60° orientation, (**i**,**j**) 70° orientation (unit: meter).

**Figure 6:**
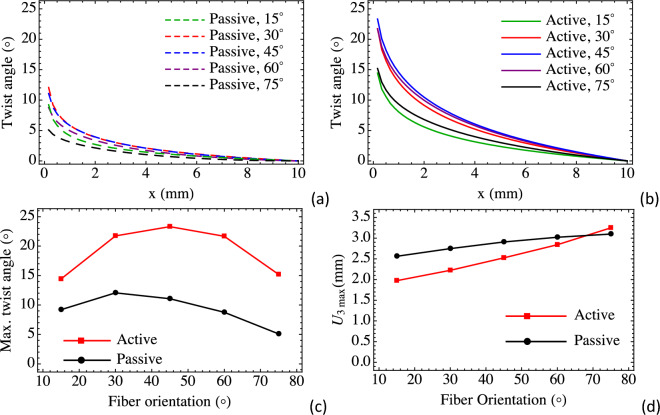
Twist angles of the horizontal centerline for (**a**) passive and (**b**) active cases, (**c**) peak twist angles for various order (fiber) orientations, and (**d**) magnitudes of peak deflections.

To achieve and amplify complex shape actuation, we considered a distribution order oriented at angles 15°, 30°, 45°, 60°, and 75° from the radial direction. We demonstrate the ability to achieve significant surface twist originating from the intrinsic material anisotropy, and to more than double the twist angle with activation. Figure 5 shows the deformed profiles predicted by our computational simulations. The deformed centerline is overlaid with the contour to demonstrate the twist. Overall, although passively anisotropic materials in this configuration (left column) will twist, this motion is enhanced by activation (right column). Figure 6 reports out the twist angles of the horizontal centerline (0–10 mm due to the symmetry) for passive and active loading. The maximum twist occurs at the center of the circular membrane and range from 14° to 24° for the active case and from 5° to 13° for the passive case. For fiber angles smaller than 75° the peak deflections of the deformed active configurations are smaller than that of the deformed passive configurations, however, the peak deflections of the deformed active configurations are greater than that of the deformed passive configurations for order angle: 75°, which is consistent with results in Fig. 4 that radial order (0°) reduces the peak deflection whilst circumferential order (90°) increases the peak deflection.

The above simulations demonstrate how we can program three-dimensional surface conformations (magnitudes, slopes, curvatures, and peak deflections etc.) via active material anisotropy starting with a single initial design. With this new mode of activation, the surface curvature and the direction of actuation can be altered (controlled). The results also demonstrate how to break the deformation symmetry via spatial activation. In addition, the simulations highlight a new membrane actuation mode: active surface twist due to intrinsic anisotropy. This motion is comparable to the twisting motion of the natural heart tissue when contracting. Interestingly enough, although the individual contractility of myocardial muscle fibers is 15–20%, studies show that the ejection fraction is 60–70% owing to fiber orientation effects^47^. In Ref.^10^, a soft bioinspired material system inspired by the geometry and mechanical functionality of the natural heart demonstrated twisting. The system was fabricated by embedding McKibben actuators as macroscopic fibers in a soft elastomer and actuated by pneumatically activating the oriented fibers. Although we have a long way to go to achieve true myocardial functionality, leveraging surface twist in soft active materials might offer exciting possibilities for soft robotics.

### Complex material architectures

#### Patterning intrinsic anisotropy

Inspired by the biological wing structure of bats, we patterned membranes with a combination of active and passive regions to illustrate the concept of spatially distributed activation in a discrete sense. In so doing, the results show how activation changes the curvature of the surface due to stretching of the membrane.

**Figure 7:**
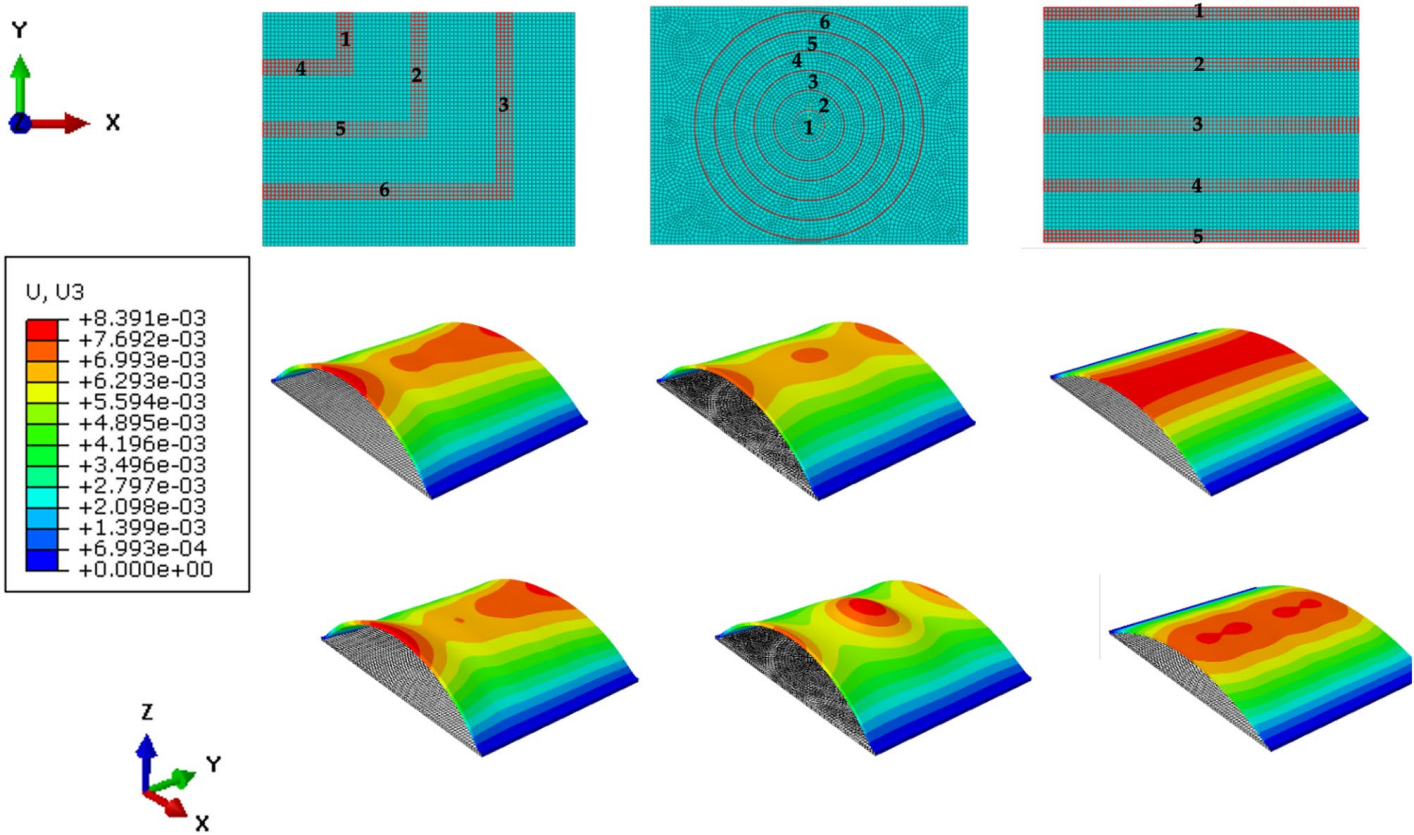
Simulation results showing surface conformations: unactuated and actuated for three different material architectures.

Consider a rectangular membrane with the following dimensions: length 40 mm, width 30 mm, and thickness 0.5 mm. The short sides of the membrane are pinned while the longer sides are free. A pressure of 50 Pa is applied on the bottom surface. The magnitude for the electric field in the fibers is set at 10 MV/m for all the simulations. We consider three material architectures representing discrete active zones (Fig. 7—top row). Simulation results show the mechanical response of the passive anisotropic membrane with just a bias pressure applied (middle figures) together with the electromechanical response of the membrane (bottom figures). The results show that nonuniform positive and negative Gaussian curvatures can be achieved and drastically enhanced by activation due to the electromechanical coupling.

## Summary

Inspired by soft active materials in biology and engineering and the potential for life-like motion with soft robotic materials, we developed a computational framework for simulating the response of soft intrinsically-anisotropic active materials. We developed a constitutive model to describe the activation of a soft material containing contractile units. The model itself is constructed within a nonlinear continuum mechanics framework for inelastic materials undergoing finite deformations. In this paper, we carried out extensive computational analysis that highlight novel mechanisms that could be exploited to develop new soft robotic materials and soft robotic actuators. We introduced three new concepts: (i) dual mode activation via out of plane matrix activation and in-plane ‘fiber’ activation due to molecular order, (ii) a surface twisting mode due to material anisotropy (both passive and active), and (iii) sequential and spatial activation to obtain distinct 3D surface conformations. The role of directionality in influencing membrane shape is emphasized. Novel highlights include complex shapes such as symmetry-breaking surface conformations, negative Gaussian curvatures, and the ability to control deflection and direction of curvature with a single initial design. Ultimately, the intrinsic material architecture could be used to obtain complex shapes such as that of a human face and dynamically altered.

The work is computational in nature only. Our computational formulation is a useful numerical tool to explore various material architectures and actuator designs with complex intrinsic anisotropy of arbitrary geometry. This ultimately plays an important role in advancing the area of soft materials research. The results point to novel concepts for future development of soft active materials and the model is a tool on that path. There is a real challenge in the synthesis, fabrication, manufacturing and development of advanced novel materials that can dynamically and reliably change shape via fast activation. New materials such as dielectric liquid crystal elastomers might offer one such opportunity. Yet still synthetic biology and biotechnology might quickly rise to deliver engineered living materials, whereby living muscle cells and living organisms might be the active and functional constituents within a host synthetic material^48^. New chemistries, processing and manufacturing technologies could be advanced to fabricate soft materials with spatially varying intrinsic anisotropy^49^. Once manufactured, additional actuation degrees of freedom could be conferred via patterning as has been recently demonstrated in photothermally activated LCEs. Experimentally, it is expected that material and structural incompatibilities might arise and alter the target response. This may be a focus of future studies. In terms of design, it is tempting to think about inverse modeling tools, however, new machine learning tools could be a powerful approach to efficiently design material architectures for target responses. This might be another future direction.

## Supplementary Information

Supplementary Video 1.

Supplementary Information 1.

Supplementary Information 2.

Supplementary Information 3.

## Data Availability

The numerical code used in this study is provided in the Supplementary Materials.
