# Supplementary material for: Methods for numerical simulation of soft actively contractile materials
Source: Sci Rep. 2023 Jun 26;13:10369. doi: 10.1038/s41598-023-36465-x (PMC10293255; doi:10.1038/s41598-023-36465-x)
Supplement: Supplementary file 2 — Supplementary Information 1. [file 41598_2023_36465_MOESM2_ESM.docx]

**Supplementary Material A**

**Kinematics**

Consider a typical material point in the reference configuration $\boldsymbol{X}$, where $\boldsymbol{\chi}$ is the macroscopic motion that maps to a position in the current configuration. The deformation gradient is defined as

$\boldsymbol{F}=\frac{\partial\boldsymbol{\chi}}{\partial\boldsymbol{X}}$ . (1)

In finite viscoelasticity, we apply a multiplicative decomposition of the deformation gradient into elastic and viscous parts,

$\boldsymbol{F}=\boldsymbol{F}_{F}^{e}\boldsymbol{F}_{F}^{v}$, (2)

where $\boldsymbol{F}_{F}^{e}$ and $\boldsymbol{F}_{F}^{v}$ are elastic and viscous parts of the fiber deformation gradient, respectively. The right and left Cauchy-Green tensors can then be defined as

$\boldsymbol{C}=\boldsymbol{F}^{T}\boldsymbol{F}, \boldsymbol{C}_{F}^{e}=\boldsymbol{F}_{F}^{eT}\boldsymbol{F}_{F}^{e}, \boldsymbol{C}_{F}^{v}=\boldsymbol{F}_{F}^{vT}\boldsymbol{F}_{F}^{v}$

$\mathbf{b}=\mathbf{FF}^{T}$ (3)

**Coupled Constitutive Equations**

For a material with intrinsic anisotropy, a directional vector, ***V***, can be used to describe the preferential orientation arising from the microstructure. If there are 2 electro-responsive directors, we have 12 possible invariants [52]. For a viscoelastic material with one fiber family, we have the free energy function with functional dependence

$\Psi\left( \boldsymbol{F}, \boldsymbol{F}_{F}^{v},\boldsymbol{V},\boldsymbol{E} \right)=\bar{\Psi}(I_{1}\sim I_{4},I_{1}^{v}\sim I_{4}^{v},I_{10}\sim I_{12}),$ (4)

where ***V*** is the unit fiber orientation vector, ***E*** is the nominal electric field, and  *I_i_* are the principal invariants. We ignore any potential dependence on cross-coupling terms and renumber the invariants for continuity. The definitions are summarized in Figure 1.

| 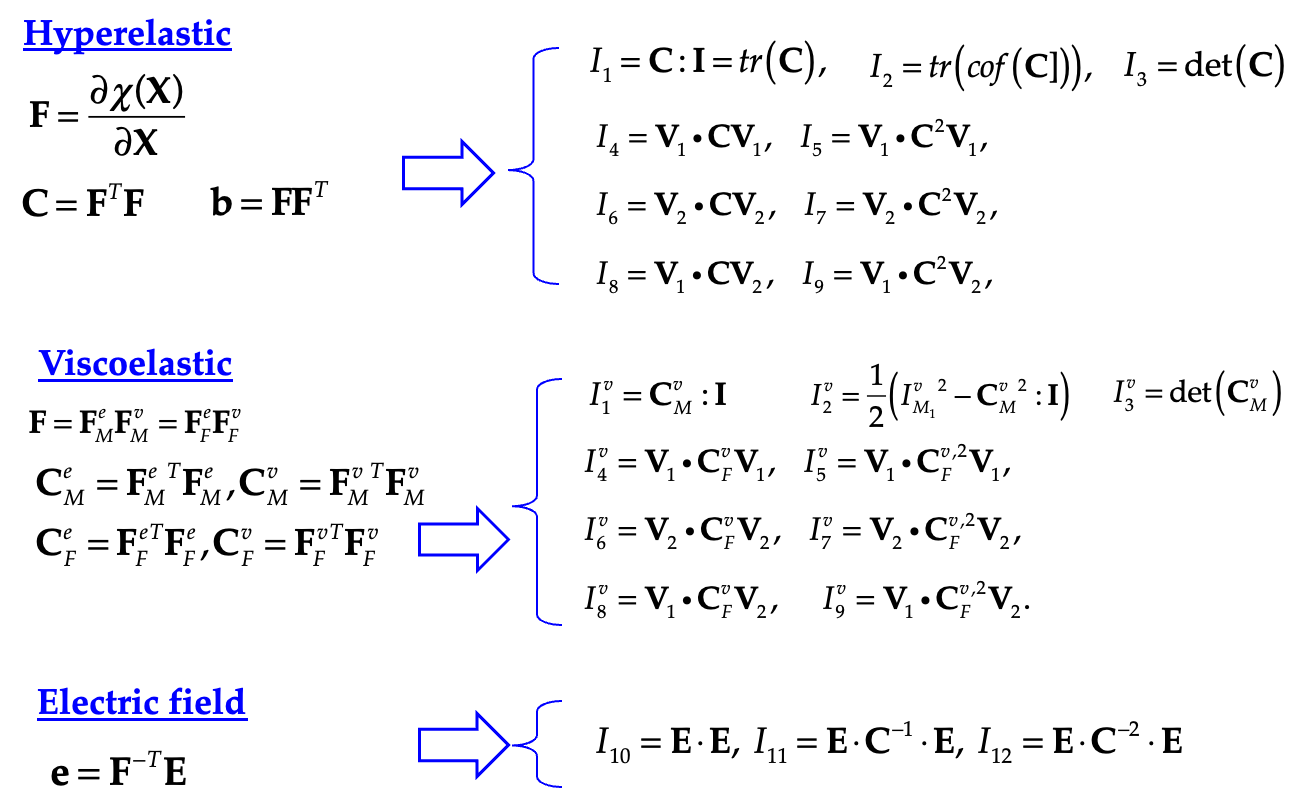 |
| --- |
| Supplementary Figure 1. Summary of invariant definitions. |

For an isotropic material, the principal invariants are expressed in terms of the right Cauchy-Green tensor as

, (5)

where $I_{1},I_{2},I_{3}$ represent the length, area, and volume change of the solid; *tr*, *cof*, *det* are the trace, cofactor, and determinant of the right Cauchy-Green tensor, respectively. In the current configuration, we have **v**=**FV**. Following Spencer [50-55], the principal invariant for the contractile fibers in the composite matrix are

, (6)

where $I_{4}$ is the square of the fiber stretch. The relationship between the viscous and elastic parts is

. (7)

The normalized fiber orientation vector in the current configuration is

. (8)

The principal invariants associated with the electric field, are defined as

(9)

The invariants $I_{5}$ and $I_{6}$ represent the quadratics of the nominal and true electric field ***E*** and ***e***. The components of the applied electric field and the unit fiber orientation vector given by

and . (10)

We then apply an electric field in the fiber direction as

. (11)

This is the magnitude of the nominal electric field in the fiber direction. Therefore, we can write the electric field activation of the matrix and of the fibers as

, (12)

and rewrite $I_{6}$ in terms of matrix and fiber parts

, (13)

where $\lambda$ is the fiber stretch and therefore $E_{f}/\lambda$ is the true electric field in the fiber.

Explicit Functional Forms: Matrix and Fiber Activation

For simplicity, we employ a NeoHookean model for the matrix and assume a quadratic model for the equilibrium and nonequilibrium fiber parts of the strain energy. The electromechanical coupling is modeled by a quadratic dependence on the true electric field. Explicit forms of both the assumed strain energy and Cauchy stress formulations are then

(14)

(15)

where $\mu$ is the shear modulus of the matrix, **b** is the left Cauchy Green tensor, $\mu_{feq}$ and $\mu_{fneq}$ are the moduli for the equilibrium and nonequilibrium parts of the fiber modulus part, $I_{4}$ is the square of the fiber stretch, $I_{4}^{v}$ is the viscous part of the square of the fiber stretch, **v** defines the orientation of the contractile units, $\varepsilon_{rf}$ and $\varepsilon_{rm}$ are the fiber and matrix dielectric constants, $\varepsilon_{0}$ is vacuum permittivity, $E_{f}$ is the nominal fiber electric field, $\boldsymbol{e}_{m}$ is the matrix true electric field, **I** is the identity tensor, and *p* is the hydrostatic pressure. The true electric field for the fiber part is $E_{f}/\lambda$ , the true electric field for the isotropic matrix is $\boldsymbol{e}_{m}=\boldsymbol{F}^{-T}\boldsymbol{E}_{m}$, and $\text{v}/\sqrt{I_{4}}$ is the unit fiber vector in the current configuration. In the absence of an electric field in the fiber direction, the familiar Maxwell stress tensor is recovered, and the stress expression in **Eq. 15** recovers the anisotropic formulation for dielectric elastomers (16)

In the absence of embedded fibers, the stress in **Eq. 15** is reduced to the familiar variation for isotropic dielectric elastomers

. (17)

Explicit Functional Forms: Evolution of contractile activation

Finite inelasticity requires an evolution equation governing the internal variable $I_{4}^{v},$ which needs to satisfy the Clausius-Duhem inequality derived from the second law of thermodynamics. Here, we employ the following linear form

, (18)

. (19)

where $\Psi$ is the Helmholtz free-energy function, $\tau_{neq}$ is the nonequilibrium stress driving the internal variable, $\eta$ is a viscous parameter, and $\dot{I}_{4}^{v}$ is the viscous fiber stretch rate. This form has been widely adopted to model viscous effects in soft materials including biological muscle. The essential equations for the model formulation is summarized in **Table 1**.

**Table 1**. Summary of final model equations.

| Strain energy function:  Cauchy stress:    Evolution equation: |
| --- |

**Computational Implementation in the Commercial FEM software ABAQUS**

In ABAQUS there are two ways to introduce a user material, one is through a user defined element and the other is through a user defined material or UMAT. To define an incompressible material in ABAQUS, we decompose the kinematic equations, stress quantities, and tangent modulus into isochoric and volumetric parts and set the bulk modulus in the volumetric part to be a very large value (normally $10^{4}$~$10^{6}$ times of the shear modulus) to simulate the almost incompressible condition.

For the UMAT, we start with the tangent modulus in the material description and decompose the total tangent modulus into isochoric and volumetric parts as

, (20)

where $\boldsymbol{S}$ is the second Piola Kirchoff stress, $\Psi_{iso}$ and $\Psi_{vol}$ are the isochoric and volumetric parts of the mechanical component of the total strain energy function, and and are the isochoric and volumetric parts of the tangent modulus. Since the strain energy function is in terms of principal invariants, the fictitious tangent modulus in the material description is defined as

, (21)

where $\delta_{1}=4\left(\frac{\partial^{2}\Psi_{iso}}{\partial\bar{I}_{1}\partial\bar{I}_{1}}\right)$, $\delta_{5}=4\left(\frac{\partial^{2}\Psi_{iso}}{\partial\bar{I}_{1}\partial\bar{I}_{4}}\right)$, $\delta_{7}=4\frac{\partial^{2}\Psi_{iso}}{\partial\bar{I}_{4}\partial\bar{I}_{4}}$ , $\mathbf{M}=\mathbf{V}\otimes\mathbf{V}$, and the isochoric part of the mechanical component of the strain energy function as

$\Psi_{iso}=\frac{\mu}{2}\left(\bar{I}_{1}-3\right)+\frac{\mu_{feq}}{2}\left(\bar{I}_{4}-1\right)^{2}+\frac{\mu_{fneq}}{2}\left(\frac{\bar{I}_{4}}{I_{4}^{v}}-1\right)$. (22)

The isochoric part of the tangent modulus in the material description is

(23)

Next, plug **Eq 21** in the double contractions in **Eq 23** and applying the general relation

$\left(\mathbf{A}\otimes\mathbf{B}\right):\left(\mathbf{C}\otimes\mathbf{D}\right)=\left(\mathbf{B}:\mathbf{C}\right)\left(\mathbf{A}\otimes\mathbf{D}\right)=tr\left(\mathbf{BC}\right)\left(\mathbf{A}\otimes\mathbf{D}\right)$, (24)

we have

(25)

Further reduction is taken by using $tr\left(\mathbf{C}\right)=I_{1}$ and $\mathbf{C}:\mathbf{M}=I_{4}$ to get the isochoric part of the tangent modulus as

(26)

The isochoric part of the tangent modulus in the spatial description is then obtained by operating the push-forward on the isochoric part of the tangent modulus in the material description in **Eq 26** as

(27)

The volumetric part of the mechanical component of the strain energy function takes the general form

$\Psi_{vol}=K\gamma^{-2}\left(\gamma LnJ+J^{-\gamma}-1\right)$, (28)

and in the current work, we use $\gamma=-1$ in **Eq 28** and other values of $\gamma$in the above formulation are optional. The volumetric part of the tangent modulus in the spatial description is

(29)

where $p=\frac{d\Psi_{vol}}{dJ}=\frac{1}{J}K\left(J-1\right)$. Finally, the total tangent modulus in the spatial description is the sum of the isochoric and volumetric tangent modulus in **Eqs 27** and **29** as

. (30)

Together with **Eqs 27** and **29**, the ABAQUS tangent modulus in the spatial description is thus

. (31)

The UMAT can be readily modified for other particularized constitutive equations.

**FEM code Verification**

Whilst solutions for simple cases can be obtained by hand, it becomes unwieldly for complex geometric configurations and complicated boundary conditions. We implemented the model using the UMAT user subroutine in ABAQUS. We verified the finite element implementation of the model by comparing with our analytical solutions for uniaxial, biaxial, and simple shear. We consider fibers aligned in the x direction for uniaxial, biaxial, and the first simple shear tests; and fibers oriented in the y direction for the second simple shear test. Analytical solutions are derived with a constant electric field magnitude of 10 MV/m along the fibers. The deformation gradients are

, ,

for uniaxial, equibiaxial, and simple shear tests, respectively. We set the stretch rate to be roughly equivalent to quasi-static (0.001/s) and use the numerical values in **Table 2** for the remaining parameters.

Table 2. Numerical values for material parameters used to check the FEM code.

| Parameters | (kPa) |  |  | (s) | (F/m) |  |  |
| --- | --- | --- | --- | --- | --- | --- | --- |
| Values | 10 | 1 | 1 | 0.25 | 8.85E-12 | 4.7 | 1 |

Consider a unit cube subjected to uniaxial, equibixial, and simple shear tests (**Fig. 6** (left column)). The FEM model is meshed using one element to eliminate error from element to element interaction. The analytical stress-stretch curves for the three typical tests are presented in **Supplementary Fig. 2** (right column) overlaid with ABAQUS FEM results. The computational results and the analytical results agree very well with each other.

| (a)(b)  (c)(d)  (e)(f)  (g)(h) |
| --- |
| **Supplementary Fig. 2** Comparison of FEM results and analytical solutions for: uniaxial loading (a-b), equibiaxial loading (c-d), and simple shear loadings (e-h). In (e-f) fibers are in the x-direction, and in (g-h) fibers are in the y-direction. |
