# Supplementary material for: Methods for numerical simulation of soft actively contractile materials
Source: Sci Rep. 2023 Jun 26;13:10369. doi: 10.1038/s41598-023-36465-x (PMC10293255; doi:10.1038/s41598-023-36465-x)
Supplement: Supplementary file 3 — Supplementary Information 2. [file 41598_2023_36465_MOESM3_ESM.docx]

**Supplementary Material B**

**Time series effects**

Programming the activation procedure creates distinct surface conformations with a single initial design. Table 1 lists the activation procedures (P1:P4) used to generate results that highlight time series effects for the material architecture in Figure 1 (a). For sequential activation of the five zones (P2), we consider a total activation time of 10 seconds with a 2 second time interval between activating any two zones in the sequence. This means that each activated zone goes to steady state before the next zone is activated considering the characteristic relaxation time of the material is 0.25 s. We plot the transverse deflection of the centerline (at y=0), which highlights the shape conformations that can be realized using this activation procedure. Figure 1 (b) shows the evolution of the transverse deflections of the centerline (at y=0) for various activation procedures. The transient time for each activation is less than 1 second ($\bar{\eta}=0.25s)$. Additionally, we simulate results for a material response time of $\bar{\eta}=2.5s$ using P2, i.e. activating zones 1~5 zones sequentially with a time interval between activating any two zones of 2 seconds (Figures 1 (c)-(f)). The transient time for each activation is longer than 2 seconds.

**Table 1.** Activation procedures for the results shown in Fig. 1.

| **Activation procedure** | **Label** |
| --- | --- |
| 1,2,3,4, and 5 zones activated at the same time | P1 |
| 1,2,3,4, and 5 zones activated sequentially | P2 |
| Only 1,3, and 5 zones activated at the same time | P3 |
| Only 2 and 4 zones activated at the same time | P4 |


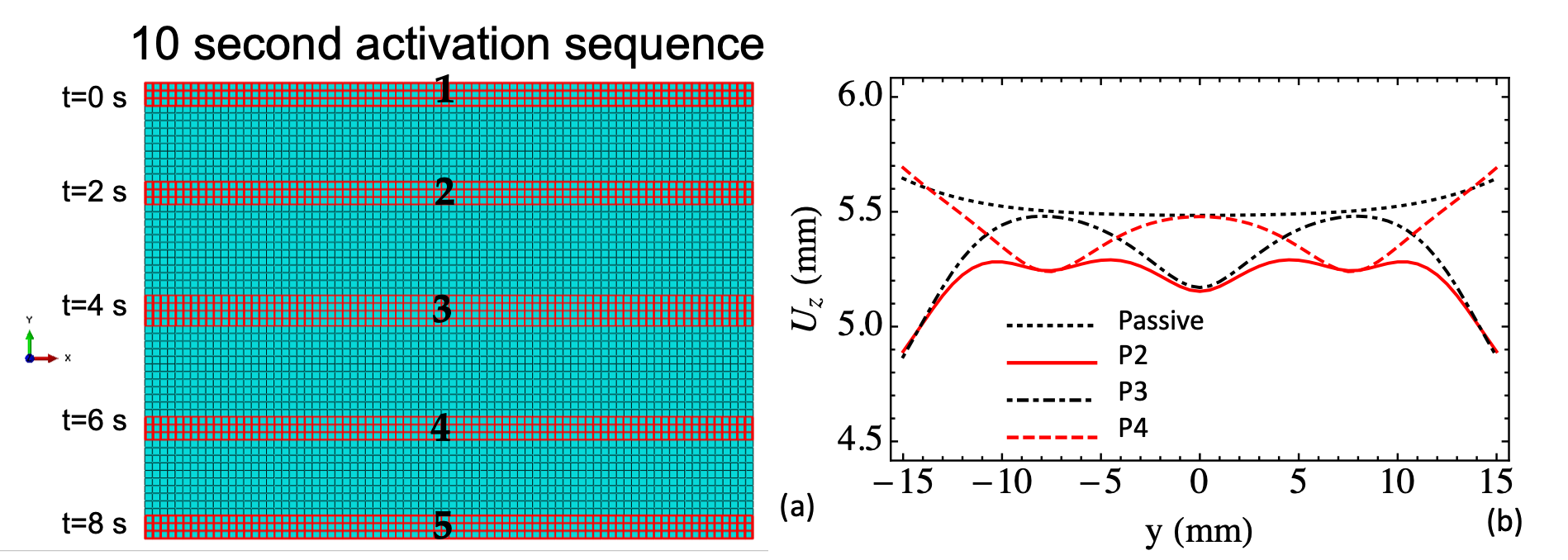


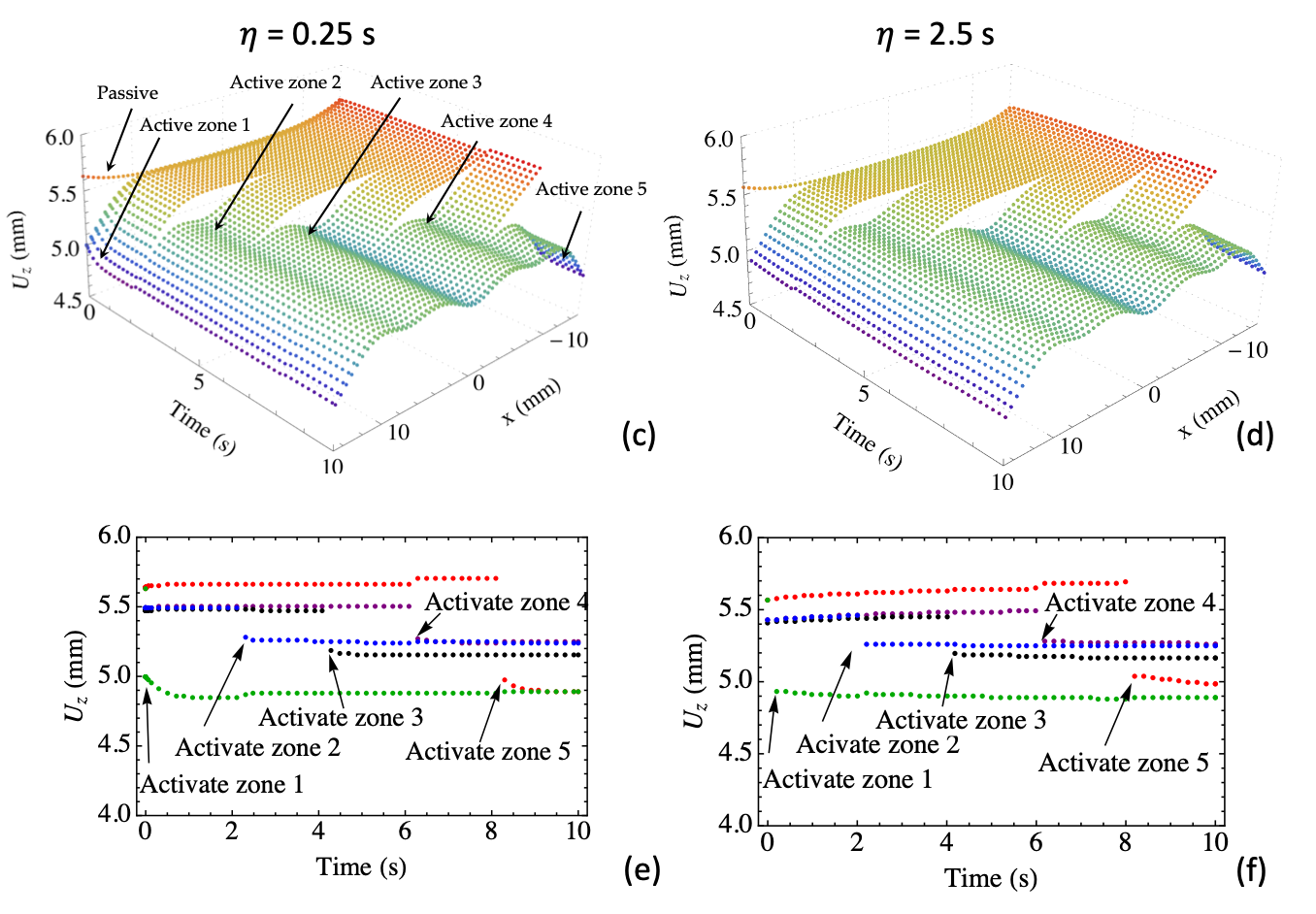


**Figure 1**. (Top) (a) Intrinsic material architecture, (b) transverse deflections of the centerlines along y-direction (at x=0 mm) for various activation procedures. (Middle) Evolution of the transverse deflections of the centerlines along the x-direction (at y=0) for sequentially activating zones 1~5 for 0.25s (c) and 2.5s (d). (Bottom) Overlay of peak transverse deflections of zones 1~5 at y=0 for material parameters of 0.25s (left) and 2.5s (right).
