# Supplementary material for: Methods for numerical simulation of soft actively contractile materials
Source: Sci Rep. 2023 Jun 26;13:10369. doi: 10.1038/s41598-023-36465-x (PMC10293255; doi:10.1038/s41598-023-36465-x)
Supplement: Supplementary file 4 — Supplementary Information 3. [file 41598_2023_36465_MOESM4_ESM.docx]

# UMAT for Soft Actively Contractile Materials

**UMAT for activation in fibers:**

SUBROUTINE UMAT(STRESS,STATEV,DDSDDE,SSE,SPD,SCD,

1 RPL,DDSDDT,DRPLDE,DRPLDT,

2 STRAN,DSTRAN,TIME,DTIME,TEMP,DTEMP,PREDEF,DPRED,CMNAME,

3 NDI,NSHR,NTENS,NSTATV,PROPS,NPROPS,COORDS,DROT,PNEWDT,

4 CELENT,DFGRD0,DFGRD1,NOEL,NPT,LAYER,KSPT,KSTEP,KINC)

C

INCLUDE 'ABA_PARAM.INC'

C

CHARACTER*8 CMNAME

DIMENSION STRESS(NTENS),STATEV(NSTATV),

1 DDSDDE(NTENS,NTENS),DDSDDT(NTENS),DRPLDE(NTENS),

2 STRAN(NTENS),DSTRAN(NTENS),TIME(2),PREDEF(1),DPRED(1),

3 PROPS(NPROPS),COORDS(3),DROT(3,3),DFGRD0(3,3),DFGRD1(3,3),

4 Stress_Max(6),VVE(6), VE(3),

5 TEMP(1),DTEMP(1),DFGRDP(3),DFGRDM1(3, 3),DFGRDM0(3, 3),

6 CBAR(3,3),DDSDDE_Max(6,6)

C

DIMENSION BBAR(6),DISTGR(3,3), VV(6), VB(3),

1 TTAO(NTENS), TAOBAR(NTENS), DELTA(6),DEL(3, 3)

C

PARAMETER(ZERO=0.D0, ONE=1.D0, TWO=2.D0, THREE=3.D0, FOUR=4.D0,

1 FIVE=5.D0, SIX=6.D0, SEVEN=7.D0, EIGHT=8.D0, NINE=9.D0,

2 NEWTON=20, TOLER=1.0d-4)

C ----------------------------------------------------------------

C STRAIN ENERGY FUNCTION_MECHANICAL PART

C W=mup(I-3)/2+mufeq/2*(I4-1)**2+muf/2*(I4/I4v-1)**2+k*(J-LnJ-1)

C ----------------------------------------------------------------

C PROPS(1) - mup

C ----------------------------------------------------------------

PMUP=PROPS(1)

PMUFEQ=10000

PMUF=10000

PKA=1E8

V1=0

V2=1

V3=0

ETA=1.E+4

EPSILON=2.3895E-11

C

C JACOBIAN AND DISTORTION TENSOR

C

DET=DFGRD1(1, 1)*DFGRD1(2, 2)*DFGRD1(3, 3)

1 -DFGRD1(1, 2)*DFGRD1(2, 1)*DFGRD1(3, 3)

IF(NSHR.EQ.3) THEN

DET=DET+DFGRD1(1, 2)*DFGRD1(2, 3)*DFGRD1(3, 1)

1 +DFGRD1(1, 3)*DFGRD1(3, 2)*DFGRD1(2, 1)

2 -DFGRD1(1, 3)*DFGRD1(3, 1)*DFGRD1(2, 2)

3 -DFGRD1(2, 3)*DFGRD1(3, 2)*DFGRD1(1, 1)

END IF

SCALE=DET**(-ONE/THREE)

DO K1=1, 3

DO K2=1, 3

DISTGR(K2, K1)=SCALE*DFGRD1(K2, K1)

END DO

END DO

C

C 3BY3 INDENTITY MATRIX AND 6BY1 IN VOIGT FORM

C

DEL(1,1)=ONE

DEL(2,2)=ONE

DEL(3,3)=ONE

DEL(1,2)=ZERO

DEL(1,3)=ZERO

DEL(2,3)=ZERO

DEL(2,1)=ZERO

DEL(3,1)=ZERO

DEL(3,2)=ZERO

DELTA(1)=ONE

DELTA(2)=ONE

DELTA(3)=ONE

DELTA(4)=ZERO

DELTA(5)=ZERO

DELTA(6)=ZERO

C

C CALCULATE LEFT CAUCHY-GREEN TENSOR (B=F.Ft)

C

BBAR(1)=DISTGR(1, 1)**2+DISTGR(1, 2)**2+DISTGR(1, 3)**2

BBAR(2)=DISTGR(2, 1)**2+DISTGR(2, 2)**2+DISTGR(2, 3)**2

BBAR(3)=DISTGR(3, 3)**2+DISTGR(3, 1)**2+DISTGR(3, 2)**2

BBAR(4)=DISTGR(1, 1)*DISTGR(2, 1)+DISTGR(1, 2)*DISTGR(2, 2)

1 +DISTGR(1, 3)*DISTGR(2, 3)

IF(NSHR.EQ.3) THEN

BBAR(5)=DISTGR(1, 1)*DISTGR(3, 1)+DISTGR(1, 2)*DISTGR(3, 2)

1 +DISTGR(1, 3)*DISTGR(3, 3)

BBAR(6)=DISTGR(2, 1)*DISTGR(3, 1)+DISTGR(2, 2)*DISTGR(3, 2)

1 +DISTGR(2, 3)*DISTGR(3, 3)

END IF

C

C CALCULATE INVARIANT I1&I4 (WITH M BEING THE UNIT VERCTOR IN CURRENT CONFIGURATION)

C

TRBBAR=BBAR(1)+BBAR(2)+BBAR(3)

VB(1)=DISTGR(1,1)*V1+DISTGR(1,2)*V2+DISTGR(1,3)*V3

VB(2)=DISTGR(2,1)*V1+DISTGR(2,2)*V2+DISTGR(2,3)*V3

VB(3)=DISTGR(3,1)*V1+DISTGR(3,2)*V2+DISTGR(3,3)*V3

C

C CALCULATE ORIENTATION(STRUCTURAL TENSOR m)

C

VV(1)=VB(1)*VB(1)

VV(2)=VB(2)*VB(2)

VV(3)=VB(3)*VB(3)

VV(4)=VB(1)*VB(2)

VV(5)=VB(1)*VB(3)

VV(6)=VB(2)*VB(3)

TRBBARR=VV(1)+VV(2)+VV(3)

c

C SET UP ALL THE QUANTITIES TO SOLVE EVOLUTION EQUATION BY NEWTON ITERATION IN THE DO LOOP

C

ONTW=ONE/TWO

THTW=THREE/TWO

C

C SET UP THE INITIAL AND VALUES OF I4VTRIAL

C

PI4V=STATEV(1)

C

C EXPLICIT FORM TO CALCULATE I4V

C

RHS=Four*PMUF*(TRBBARR/PI4V-ONE)*TRBBARR/

1 PI4V

PI4V=PI4V+RHS*DTIME/ETA

C

C CALCULATE DW/DI AND D2W/D2I

C

WO=PMUP/TWO

WOO=FOUR*ZERO !delta1

WF=PMUFEQ*(TRBBARR-ONE)+PMUF*(TRBBARR/PI4V-ONE)/PI4V

WFF=FOUR*(PMUFEQ+PMUF/PI4V/PI4V) !delta7

WOF=FOUR*ZERO ! delat5

C

C CALCULATE THE STRESSES AND TR(TAOBAR)

C

TRBBART=TRBBAR/THREE

TRBBARRT=TRBBARR/THREE

EG=TWO/DET

PR=TWO*PKA*(DET-ONE)

PRBAR=TWO*PKA*(TWO*DET-ONE)

DO K1=1,NDI

TTAO(K1)=TWO*WO*(BBAR(K1)-TRBBART)+TWO*WF*(VV(K1)-TRBBARRT)

TAOBAR(K1)=TWO*WO*BBAR(K1)+TWO*WF*VV(K1)

STRESS(K1)=EG*WO*(BBAR(K1)-TRBBART)+EG*WF*(VV(K1)-TRBBARRT)

1 +PR

END DO

DO K1=NDI+1,NDI+NSHR

TTAO(K1)=TWO*WO*BBAR(K1)+TWO*WF*(VV(K1))

TAOBAR(K1)=TWO*WO*BBAR(K1)+TWO*WF*(VV(K1))

STRESS(K1)=EG*WO*BBAR(K1)+EG*WF*(VV(K1))

END DO

TRTAOBAR=TAOBAR(1)+TAOBAR(2)+TAOBAR(3)

C

C CALCULATE THE STIFFNESS

C

DO K2=1, 3

DO K1=1, 3

DDSDDE(K1, K2)=PRBAR*DELTA(K1)*DELTA(K2)

1 -PR*TWO*DEL(K1,K2)+(WOO*BBAR(K1)*BBAR(K2)

2 +WOF*(BBAR(K1)*VV(K2)+VV(K1)*BBAR(K2))

3 +WFF*VV(K1)*VV(K2))/DET-((WOO*TRBBAR+WOF*TRBBARR)

4 *(DELTA(K1)*BBAR(K2)+BBAR(K1)*DELTA(K2))+

5 (WOF*TRBBAR+WFF*TRBBARR)*(DELTA(K1)*VV(K2)+VV(K1)

6 *DELTA(K2)))/(THREE*DET)+(WOO*TRBBAR**TWO+

7 TWO*WOF*TRBBAR*TRBBARR+WFF*TRBBARR**TWO)*DELTA(K1)

8 *DELTA(K2)/(NINE*DET)+TRTAOBAR*(ONE/TWO*TWO*DEL(K1,K2)

9 -ONE/THREE*DELTA(K1)*DELTA(K2))*TWO/(THREE*DET)-

1 (DELTA(K1)*TTAO(K2)+TTAO(K1)*DELTA(K2))*TWO/(THREE*DET)

END DO

END DO

DO K2=4, 6

DO K1=4, 6

DDSDDE(K1, K2)=PRBAR*DELTA(K1)*DELTA(K2)

1 -PR*ONE*DEL(K1-THREE,K2-THREE)+(WOO*BBAR(K1)

2 *BBAR(K2)+WOF*(BBAR(K1)*VV(K2)+VV(K1)*BBAR(K2))

3 +WFF*VV(K1)*VV(K2))/DET-((WOO*TRBBAR+WOF*TRBBARR)

4 *(DELTA(K1)*BBAR(K2)+BBAR(K1)*DELTA(K2))+

5 (WOF*TRBBAR+WFF*TRBBARR)*(DELTA(K1)*VV(K2)+VV(K1)

6 *DELTA(K2)))/(THREE*DET)+(WOO*TRBBAR**TWO+

7 TWO*WOF*TRBBAR*TRBBARR+WFF*TRBBARR**TWO)*DELTA(K1)

8 *DELTA(K2)/(NINE*DET)+TRTAOBAR*(ONE/TWO*ONE*

9 DEL(K1-THREE,K2-THREE)-ONE/THREE*DELTA(K1)*DELTA(K2))

1 *TWO/(THREE*DET)-(DELTA(K1)*TTAO(K2)+TTAO(K1)*DELTA(K2))

2 *TWO/(THREE*DET)

END DO

END DO

DO K2=4, 6

DO K1=1, 3

DDSDDE(K1, K2)=PRBAR*DELTA(K1)*DELTA(K2)

1 -PR*ZERO*DEL(K1,K2-THREE)+(WOO*BBAR(K1)*BBAR(K2)

2 +WOF*(BBAR(K1)*VV(K2)+VV(K1)*BBAR(K2))

3 +WFF*VV(K1)*VV(K2))/DET-((WOO*TRBBAR+WOF*TRBBARR)

4 *(DELTA(K1)*BBAR(K2)+BBAR(K1)*DELTA(K2))+

5 (WOF*TRBBAR+WFF*TRBBARR)*(DELTA(K1)*VV(K2)+VV(K1)

6 *DELTA(K2)))/(THREE*DET)+(WOO*TRBBAR**TWO+

7 TWO*WOF*TRBBAR*TRBBARR+WFF*TRBBARR**TWO)*DELTA(K1)

8 *DELTA(K2)/(NINE*DET)+TRTAOBAR*(ONE/TWO*ZERO*

9 DEL(K1,K2-THREE)-ONE/THREE*DELTA(K1)*DELTA(K2))*TWO

1 /(THREE*DET)-(DELTA(K1)*TTAO(K2)+TTAO(K1)*DELTA(K2))

2 *TWO/(THREE*DET)

END DO

END DO

DO K2=1, 3

DO K1=4, 6

DDSDDE(K1, K2)=PRBAR*DELTA(K1)*DELTA(K2)

1 -PR*ZERO*DEL(K1-THREE,K2)+4*(WOO*BBAR(K1)*BBAR(K2)

2 +WOF*(BBAR(K1)*VV(K2)+VV(K1)*BBAR(K2))

3 +WFF*VV(K1)*VV(K2))/DET-4*((WOO*TRBBAR+WOF*TRBBARR)

4 *(DELTA(K1)*BBAR(K2)+BBAR(K1)*DELTA(K2))+

5 (WOF*TRBBAR+WFF*TRBBARR)*(DELTA(K1)*VV(K2)+VV(K1)

6 *DELTA(K2)))/(THREE*DET)+4*(WOO*TRBBAR**TWO+

7 TWO*WOF*TRBBAR*TRBBARR+WFF*TRBBARR**TWO)*DELTA(K1)

8 *DELTA(K2)/(NINE*DET)+TRTAOBAR*(ONE/TWO*ZERO

9 *DEL(K1-THREE,K2)-ONE/THREE*DELTA(K1)*DELTA(K2))*TWO

1 /(THREE*DET)-(DELTA(K1)*TTAO(K2)+TTAO(K1)*DELTA(K2))

2 *TWO/(THREE*DET)

END DO

END DO

DDSDDE(1,1)=DDSDDE(1,1)+TWO*STRESS(1)

DDSDDE(2,2)=DDSDDE(2,2)+TWO*STRESS(2)

DDSDDE(3,3)=DDSDDE(3,3)+TWO*STRESS(3)

DDSDDE(4,4)=DDSDDE(4,4)+ONTW*(STRESS(1)+STRESS(2))

DDSDDE(5,5)=DDSDDE(5,5)+ONTW*(STRESS(1)+STRESS(3))

DDSDDE(6,6)=DDSDDE(6,6)+ONTW*(STRESS(2)+STRESS(3))

DDSDDE(1,4)=DDSDDE(1,4)+TWO*STRESS(4)

DDSDDE(1,5)=DDSDDE(1,5)+TWO*STRESS(5)

DDSDDE(2,4)=DDSDDE(2,4)+TWO*STRESS(4)

DDSDDE(2,6)=DDSDDE(2,6)+TWO*STRESS(6)

DDSDDE(3,5)=DDSDDE(3,5)+TWO*STRESS(5)

DDSDDE(3,6)=DDSDDE(3,6)+TWO*STRESS(6)

DDSDDE(4,5)=DDSDDE(4,5)+ONTW*STRESS(6)

DDSDDE(4,6)=DDSDDE(4,6)+ONTW*STRESS(5)

DDSDDE(5,6)=DDSDDE(5,6)+ONTW*STRESS(4)

DO K1=1, NTENS

DO K2=1, K1-1

DDSDDE(K1, K2)=DDSDDE(K2, K1)

END DO

END DO

En=12E6 !Electric field (MV/m)

Enn=En**TWO

TRBBARE=TRBBARR**TWO

DO K1=1,NDI+NSHR

STRESS_Max(K1)=EPSILON*Enn*VV(K1)/TRBBARE

STRESS(K1)=STRESS(K1)+STRESS_Max(K1)

END DO

C

C STATE VARIABLES:

C

STATEV(1)=PI4V

RETURN

END

**UMAT for activation in matrix:**

SUBROUTINE UMAT(STRESS,STATEV,DDSDDE,SSE,SPD,SCD,

1 RPL,DDSDDT,DRPLDE,DRPLDT,

2 STRAN,DSTRAN,TIME,DTIME,TEMP,DTEMP,PREDEF,DPRED,CMNAME,

3 NDI,NSHR,NTENS,NSTATV,PROPS,NPROPS,COORDS,DROT,PNEWDT,

4 CELENT,DFGRD0,DFGRD1,NOEL,NPT,LAYER,KSPT,KSTEP,KINC)

C

INCLUDE 'ABA_PARAM.INC'

C

CHARACTER*8 CMNAME

DIMENSION STRESS(NTENS),STATEV(NSTATV),

1 DDSDDE(NTENS,NTENS),DDSDDT(NTENS),DRPLDE(NTENS),

2 STRAN(NTENS),DSTRAN(NTENS),TIME(2),PREDEF(1),DPRED(1),

3 PROPS(NPROPS),COORDS(3),DROT(3,3),DFGRD0(3,3),DFGRD1(3,3),

4 Et(3),Stress_Max(3,3),EkEk(1),DFGRDM1_INV(3,3),En(3),

5 TEMP(1),DTEMP(1),DFGRDP(3),DFGRDM1(3, 3),DFGRDM0(3, 3),

6 CBAR(3,3),DDSDDE_Max(6,6)

C

DIMENSION BBAR(6),DISTGR(3,3), VV(6), VB(3),

1 TTAO(NTENS), TAOBAR(NTENS), DELTA(6),DEL(3, 3)

C

PARAMETER(ZERO=0.D0, ONE=1.D0, TWO=2.D0, THREE=3.D0, FOUR=4.D0,

1 FIVE=5.D0, SIX=6.D0, SEVEN=7.D0, EIGHT=8.D0, NINE=9.D0,

2 NEWTON=20, TOLER=1.0d-4)

C ----------------------------------------------------------------

C STRAIN ENERGY FUNCTION_MECHANICAL PART

C W=mup(I-3)/2+mufeq/2*(I4-1)**2+muf/2*(I4/I4v-1)**2+k*(J-LnJ-1)

C ----------------------------------------------------------------

C PROPS(1) - mup

C ----------------------------------------------------------------

C

PMUP=PROPS(1)

PMUFEQ=10000

PMUF=10000

PKA=1E8

V1=0

V2=1

V3=0

ETA=1.E+4

EPSILON=4.1595E-11

C

C JACOBIAN AND DISTORTION TENSOR

C

DET=DFGRD1(1, 1)*DFGRD1(2, 2)*DFGRD1(3, 3)

1 -DFGRD1(1, 2)*DFGRD1(2, 1)*DFGRD1(3, 3)

IF(NSHR.EQ.3) THEN

DET=DET+DFGRD1(1, 2)*DFGRD1(2, 3)*DFGRD1(3, 1)

1 +DFGRD1(1, 3)*DFGRD1(3, 2)*DFGRD1(2, 1)

2 -DFGRD1(1, 3)*DFGRD1(3, 1)*DFGRD1(2, 2)

3 -DFGRD1(2, 3)*DFGRD1(3, 2)*DFGRD1(1, 1)

END IF

SCALE=DET**(-ONE/THREE)

DO K1=1, 3

DO K2=1, 3

DISTGR(K2, K1)=SCALE*DFGRD1(K2, K1)

END DO

END DO

C

C 3BY3 INDENTITY MATRIX AND 6BY1 IN VOIGT FORM

C

DEL(1,1)=ONE

DEL(2,2)=ONE

DEL(3,3)=ONE

DEL(1,2)=ZERO

DEL(1,3)=ZERO

DEL(2,3)=ZERO

DEL(2,1)=ZERO

DEL(3,1)=ZERO

DEL(3,2)=ZERO

DELTA(1)=ONE

DELTA(2)=ONE

DELTA(3)=ONE

DELTA(4)=ZERO

DELTA(5)=ZERO

DELTA(6)=ZERO

C

C CALCULATE LEFT CAUCHY-GREEN TENSOR (B=F.Ft)

C

BBAR(1)=DISTGR(1, 1)**2+DISTGR(1, 2)**2+DISTGR(1, 3)**2

BBAR(2)=DISTGR(2, 1)**2+DISTGR(2, 2)**2+DISTGR(2, 3)**2

BBAR(3)=DISTGR(3, 3)**2+DISTGR(3, 1)**2+DISTGR(3, 2)**2

BBAR(4)=DISTGR(1, 1)*DISTGR(2, 1)+DISTGR(1, 2)*DISTGR(2, 2)

1 +DISTGR(1, 3)*DISTGR(2, 3)

IF(NSHR.EQ.3) THEN

BBAR(5)=DISTGR(1, 1)*DISTGR(3, 1)+DISTGR(1, 2)*DISTGR(3, 2)

1 +DISTGR(1, 3)*DISTGR(3, 3)

BBAR(6)=DISTGR(2, 1)*DISTGR(3, 1)+DISTGR(2, 2)*DISTGR(3, 2)

1 +DISTGR(2, 3)*DISTGR(3, 3)

END IF

C

C CALCULATE INVARIANT I1&I4 (WITH M BEING THE UNIT VERCTOR IN CURRENT CONFIGURATION)

C

TRBBAR=BBAR(1)+BBAR(2)+BBAR(3)

VB(1)=DISTGR(1,1)*V1+DISTGR(1,2)*V2+DISTGR(1,3)*V3

VB(2)=DISTGR(2,1)*V1+DISTGR(2,2)*V2+DISTGR(2,3)*V3

VB(3)=DISTGR(3,1)*V1+DISTGR(3,2)*V2+DISTGR(3,3)*V3

C

C CALCULATE ORIENTATION(STRUCTURAL TENSOR m)

C

VV(1)=VB(1)*VB(1)

VV(2)=VB(2)*VB(2)

VV(3)=VB(3)*VB(3)

VV(4)=VB(1)*VB(2)

VV(5)=VB(1)*VB(3)

VV(6)=VB(2)*VB(3)

TRBBARR=VV(1)+VV(2)+VV(3)

C

C SET UP ALL THE QUANTITIES TO SOLVE EVOLUTION EQUATION BY NEWTON ITERATION IN THE DO LOOP

C

ONTW=ONE/TWO

THTW=THREE/TWO

C

C SET UP THE INITIAL AND VALUES OF I4VTRIAL

C

PI4V=STATEV(1)

C

C EXPLICIT FORM TO CALCULATE I4V

C

RHS=Four*PMUF*(TRBBARR/PI4V-ONE)*TRBBARR/

1 PI4V

PI4V=PI4V+RHS*DTIME/ETA

C

C CALCULATE DW/DI AND D2W/D2I

C

WO=PMUP/TWO

WOO=FOUR*ZERO !delta1

WF=PMUFEQ*(TRBBARR-ONE)+PMUF*(TRBBARR/PI4V-ONE)/PI4V

WFF=FOUR*(PMUFEQ+PMUF/PI4V/PI4V) !delta7

WOF=FOUR*ZERO ! delat5

C

C CALCULATE THE STRESSES AND TR(TAOBAR)

C

TRBBART=TRBBAR/THREE

TRBBARRT=TRBBARR/THREE

EG=TWO/DET

PR=TWO*PKA*(DET-ONE)

PRBAR=TWO*PKA*(TWO*DET-ONE)

DO K1=1,NDI

TTAO(K1)=TWO*WO*(BBAR(K1)-TRBBART)+TWO*WF*(VV(K1)-TRBBARRT)

TAOBAR(K1)=TWO*WO*BBAR(K1)+TWO*WF*VV(K1)

STRESS(K1)=EG*WO*(BBAR(K1)-TRBBART)+EG*WF*(VV(K1)-TRBBARRT)

1 +PR

END DO

DO K1=NDI+1,NDI+NSHR

TTAO(K1)=TWO*WO*BBAR(K1)+TWO*WF*(VV(K1))

TAOBAR(K1)=TWO*WO*BBAR(K1)+TWO*WF*(VV(K1))

STRESS(K1)=EG*WO*BBAR(K1)+EG*WF*(VV(K1))

END DO

TRTAOBAR=TAOBAR(1)+TAOBAR(2)+TAOBAR(3)

C

C CALCULATE THE STIFFNESS

C

DO K2=1, 3

DO K1=1, 3

DDSDDE(K1, K2)=PRBAR*DELTA(K1)*DELTA(K2)

1 -PR*TWO*DEL(K1,K2)+(WOO*BBAR(K1)*BBAR(K2)

2 +WOF*(BBAR(K1)*VV(K2)+VV(K1)*BBAR(K2))

3 +WFF*VV(K1)*VV(K2))/DET-((WOO*TRBBAR+WOF*TRBBARR)

4 *(DELTA(K1)*BBAR(K2)+BBAR(K1)*DELTA(K2))+

5 (WOF*TRBBAR+WFF*TRBBARR)*(DELTA(K1)*VV(K2)+VV(K1)

6 *DELTA(K2)))/(THREE*DET)+(WOO*TRBBAR**TWO+

7 TWO*WOF*TRBBAR*TRBBARR+WFF*TRBBARR**TWO)*DELTA(K1)

8 *DELTA(K2)/(NINE*DET)+TRTAOBAR*(ONE/TWO*TWO*DEL(K1,K2)

9 -ONE/THREE*DELTA(K1)*DELTA(K2))*TWO/(THREE*DET)-

1 (DELTA(K1)*TTAO(K2)+TTAO(K1)*DELTA(K2))*TWO/(THREE*DET)

END DO

END DO

DO K2=4, 6

DO K1=4, 6

DDSDDE(K1, K2)=PRBAR*DELTA(K1)*DELTA(K2)

1 -PR*ONE*DEL(K1-THREE,K2-THREE)+(WOO*BBAR(K1)

2 *BBAR(K2)+WOF*(BBAR(K1)*VV(K2)+VV(K1)*BBAR(K2))

3 +WFF*VV(K1)*VV(K2))/DET-((WOO*TRBBAR+WOF*TRBBARR)

4 *(DELTA(K1)*BBAR(K2)+BBAR(K1)*DELTA(K2))+

5 (WOF*TRBBAR+WFF*TRBBARR)*(DELTA(K1)*VV(K2)+VV(K1)

6 *DELTA(K2)))/(THREE*DET)+(WOO*TRBBAR**TWO+

7 TWO*WOF*TRBBAR*TRBBARR+WFF*TRBBARR**TWO)*DELTA(K1)

8 *DELTA(K2)/(NINE*DET)+TRTAOBAR*(ONE/TWO*ONE*

9 DEL(K1-THREE,K2-THREE)-ONE/THREE*DELTA(K1)*DELTA(K2))

1 *TWO/(THREE*DET)-(DELTA(K1)*TTAO(K2)+TTAO(K1)*DELTA(K2))

2 *TWO/(THREE*DET)

END DO

END DO

DO K2=4, 6

DO K1=1, 3

DDSDDE(K1, K2)=PRBAR*DELTA(K1)*DELTA(K2)

1 -PR*ZERO*DEL(K1,K2-THREE)+(WOO*BBAR(K1)*BBAR(K2)

2 +WOF*(BBAR(K1)*VV(K2)+VV(K1)*BBAR(K2))

3 +WFF*VV(K1)*VV(K2))/DET-((WOO*TRBBAR+WOF*TRBBARR)

4 *(DELTA(K1)*BBAR(K2)+BBAR(K1)*DELTA(K2))+

5 (WOF*TRBBAR+WFF*TRBBARR)*(DELTA(K1)*VV(K2)+VV(K1)

6 *DELTA(K2)))/(THREE*DET)+(WOO*TRBBAR**TWO+

7 TWO*WOF*TRBBAR*TRBBARR+WFF*TRBBARR**TWO)*DELTA(K1)

8 *DELTA(K2)/(NINE*DET)+TRTAOBAR*(ONE/TWO*ZERO*

9 DEL(K1,K2-THREE)-ONE/THREE*DELTA(K1)*DELTA(K2))*TWO

1 /(THREE*DET)-(DELTA(K1)*TTAO(K2)+TTAO(K1)*DELTA(K2))

2 *TWO/(THREE*DET)

END DO

END DO

DO K2=1, 3

DO K1=4, 6

DDSDDE(K1, K2)=PRBAR*DELTA(K1)*DELTA(K2)

1 -PR*ZERO*DEL(K1-THREE,K2)+4*(WOO*BBAR(K1)*BBAR(K2)

2 +WOF*(BBAR(K1)*VV(K2)+VV(K1)*BBAR(K2))

3 +WFF*VV(K1)*VV(K2))/DET-4*((WOO*TRBBAR+WOF*TRBBARR)

4 *(DELTA(K1)*BBAR(K2)+BBAR(K1)*DELTA(K2))+

5 (WOF*TRBBAR+WFF*TRBBARR)*(DELTA(K1)*VV(K2)+VV(K1)

6 *DELTA(K2)))/(THREE*DET)+4*(WOO*TRBBAR**TWO+

7 TWO*WOF*TRBBAR*TRBBARR+WFF*TRBBARR**TWO)*DELTA(K1)

8 *DELTA(K2)/(NINE*DET)+TRTAOBAR*(ONE/TWO*ZERO

9 *DEL(K1-THREE,K2)-ONE/THREE*DELTA(K1)*DELTA(K2))*TWO

1 /(THREE*DET)-(DELTA(K1)*TTAO(K2)+TTAO(K1)*DELTA(K2))

2 *TWO/(THREE*DET)

END DO

END DO

DDSDDE(1,1)=DDSDDE(1,1)+TWO*STRESS(1)

DDSDDE(2,2)=DDSDDE(2,2)+TWO*STRESS(2)

DDSDDE(3,3)=DDSDDE(3,3)+TWO*STRESS(3)

DDSDDE(4,4)=DDSDDE(4,4)+ONTW*(STRESS(1)+STRESS(2))

DDSDDE(5,5)=DDSDDE(5,5)+ONTW*(STRESS(1)+STRESS(3))

DDSDDE(6,6)=DDSDDE(6,6)+ONTW*(STRESS(2)+STRESS(3))

DDSDDE(1,4)=DDSDDE(1,4)+TWO*STRESS(4)

DDSDDE(1,5)=DDSDDE(1,5)+TWO*STRESS(5)

DDSDDE(2,4)=DDSDDE(2,4)+TWO*STRESS(4)

DDSDDE(2,6)=DDSDDE(2,6)+TWO*STRESS(6)

DDSDDE(3,5)=DDSDDE(3,5)+TWO*STRESS(5)

DDSDDE(3,6)=DDSDDE(3,6)+TWO*STRESS(6)

DDSDDE(4,5)=DDSDDE(4,5)+ONTW*STRESS(6)

DDSDDE(4,6)=DDSDDE(4,6)+ONTW*STRESS(5)

DDSDDE(5,6)=DDSDDE(5,6)+ONTW*STRESS(4)

DO K1=1, NTENS

DO K2=1, K1-1

DDSDDE(K1, K2)=DDSDDE(K2, K1)

END DO

END DO

C Calculate the inverse of deformation gradient

C

DFGRDM1_INV(1,1)=DFGRD1(2,2)*DFGRD1(3,3)

1 -DFGRD1(2,3)*DFGRD1(3,2)

DFGRDM1_INV(1,2)=-DFGRD1(1,2)*DFGRD1(3,3)

1 +DFGRD1(1,3)*DFGRD1(3,2)

DFGRDM1_INV(1,3)=DFGRD1(1,2)*DFGRD1(2,3)

1 -DFGRD1(1,3)*DFGRD1(2,2)

DFGRDM1_INV(2,1)=-DFGRD1(2,1)*DFGRD1(3,3)

1 +DFGRD1(2,3)*DFGRD1(3,1)

DFGRDM1_INV(2,2)=DFGRD1(1,1)*DFGRD1(3,3)

1 -DFGRD1(1,3)*DFGRD1(3,1)

DFGRDM1_INV(2,3)=-DFGRD1(1,1)*DFGRD1(2,3)

1 +DFGRD1(1,3)*DFGRD1(2,1)

DFGRDM1_INV(3,1)=DFGRD1(2,1)*DFGRD1(3,2)

1 -DFGRD1(2,2)*DFGRD1(3,1)

DFGRDM1_INV(3,2)=-DFGRD1(1,1)*DFGRD1(3,2)

1 +DFGRD1(1,2)*DFGRD1(3,1)

DFGRDM1_INV(3,3)=DFGRD1(1,1)*DFGRD1(2,2)

1 -DFGRD1(1,2)*DFGRD1(2,1)

DO I=1,3

DO J=1,3

DFGRDM1_INV(I,J)=DFGRDM1_INV(I,J)/DET

END DO

END DO

C Nominal electric field vector

En(1)=0

En(2)=0

En(3)=5E6

DO I=1,3

Et(I)=0

END DO

DO I=1,3

DO J=1,3

Et(J)=Et(J)+En(I)*DFGRDM1_INV(I,J)

END Do

END Do

EkEk=0.0D0

DO I=1, 3

EkEk=EkEk+Et(I)*Et(I)

ENDDO

DO I=1,3

DO J=1,3

Stress_Max(I,J)=0.0D0

ENDDO

ENDDO

C

C Update the true stress due to polarization

C

DO I=1,3

DO J=1,3

IF (I==J) THEN

Stress_Max(I,J)=EPSILON*Et(I)*Et(J)-0.50D0*EPSILON*EkEk(1)

ELSE

Stress_Max(I,J)=EPSILON*Et(I)*Et(J)

ENDIF

ENDDO

ENDDO

STRESS(1)=STRESS(1)+Stress_Max(1,1)

STRESS(2)=STRESS(2)+Stress_Max(2,2)

STRESS(3)=STRESS(3)+Stress_Max(3,3)

STRESS(4)=STRESS(4)+Stress_Max(1,2)

STRESS(5)=STRESS(5)+Stress_Max(1,3)

STRESS(6)=STRESS(6)+Stress_Max(2,3)

C

C STATE VARIABLES:

C

STATEV(1)=PI4V

RETURN

END
